# Towards an Integrative Framework of Self-Regulation: A Scoping Review on the Interplay Between Emotion Regulation, Executive Functions and Decision-Making in Clinical Populations

**DOI:** 10.3390/brainsci16070702

**Published:** 2026-06-30

**Authors:** Francesca Colombi, Giulia Fusi, Maura Crepaldi, Maria Luisa Rusconi

**Affiliations:** Department of Human and Social Sciences, University of Bergamo, 24129 Bergamo, Italy; francesca.colombi@unibg.it (F.C.); giulia.fusi@unibg.it (G.F.); maura.crepaldi@unibg.it (M.C.)

**Keywords:** emotion regulation, decision-making, executive functions, dementia, traumatic brain injury, autism spectrum disorder, substance abuse, behavioural addictions, cognitive flexibility, inhibitory control

## Abstract

Background/Objective: Emotion regulation (ER) is closely linked to decision-making (DM). Although executive functions (EF) are often suggested as a key mechanism underlying this relationship, evidence from different clinical conditions remains fragmented. This scoping review aims to map the literature on the relationship between ER and DM in clinical populations, with a specific focus on the interaction between EF and ER in shaping adaptive DM processes, particularly in populations characterised by cognitive impairment and emotional dysregulation. Methods: A search of electronic databases was conducted to identify empirical studies examining ER, EF and DM in clinical populations. Sixteen studies involving patients with dementia, traumatic brain injury, autism spectrum disorder, substance and behavioural addictions were included. Results: Difficulties in ER were associated with altered DM. EF, particularly inhibitory control, working memory, and cognitive flexibility, were found to be key processes linking emotional states to decision outcomes. Clinical groups consistently showed a tendency to prefer immediate rewards despite potential long-term negative consequences and experienced difficulties integrating emotional and cognitive information. Conclusions: This scoping review emphasises the importance of moving beyond reductionist explanations of DM and to adopt an integrative approach. ER and EF should be conceptualised as interacting components of a broader self-regulatory system shaping decision behaviour. Developing this framework will be crucial to enable targeted clinical and neurorehabilitation interventions for individuals experiencing impaired DM.

## 1. Introduction

Decision-making (DM) can be conceptualised as a structured process through which individuals evaluate alternatives and select a course of action in relation to their goals and the demands of the situation [1]. Traditionally, the study of DM has been influenced by economic models that aim to identify the best choice based on a rational evaluation of outcomes [2]. However, these models often overlook emotional, social and contextual factors, which are now recognised as central to real-world DM processes. Indeed, increasing evidence indicates that actual human behaviour often diverges from purely rational predictions due to the pervasive influence of emotions [3]. One influential framework that accounts for this interaction is the dual-process theory. This theory posits that DM arises from the interplay between two systems: an affective system that is fast, automatic and emotion-driven and a deliberative system that is slower, more controlled and cognitively demanding [4]. These systems operate in parallel and may compete in shaping behavioural responses [3]. Within this framework, emotion regulation (ER) is a key mechanism through which individuals can control the influence of emotions on their decisions.

ER refers to the processes by which people influence their emotions, how they experience them and how they express them [5,6]. According to the ER process model [5,7], emotions unfold over time and can be modulated through different regulatory strategies [8]. Antecedent-focused strategies, including situation selection, situation modification, attentional deployment and cognitive reappraisal, operate before the full activation of the emotional response; response-focused strategies, such as expressive suppression, act after the emotional response has already been generated [9].

Emotions and regulation strategies can act in different ways in our decisional process, like the assessment of potential alternative options, the evaluation of a choice and the various possible outcomes [3]. The Emotion-Imbued Choice (EIC) model [10] proposes that emotions influence the entire DM process and highlights the role of current emotions at the time of choice. These emotions may arise from several factors such as dispositional traits, task characteristics, anticipatory processes, difficulty of the decision or incidental factors. Emotions shape attention, depth of processing, probability weighting, discounting, motivational goals and can also indirectly modify predicted utilities, thus influencing the perceived value of future outcomes. Emotions are thus intrinsically interconnected with DM, particularly when outcomes are personally relevant or consequential [10].

DM and ER are linked by the central role that affects play in both processes, especially in choices involving reward or punishment [11]. Experimental manipulation of ER strategies allows researchers to examine whether modulating ongoing emotional states alters subsequent choices [12]. Research has focused especially on reappraisal and suppression strategies. Reappraisal consists of modifying one’s appraisal of a situation to alter its emotional significance. By modifying appraisal early in the emotion-generative process, reappraisal attenuates the negative impact of affective responses and facilitates more adaptive cognitive processing. Suppression can influence an already existing emotional reaction inhibiting ongoing emotion-expressive behaviour [5,9]. It seems that these two strategies need different cognitive effort: reappraisal diminishes emotion at an early stage and without the need for sustained effort over time, whereas suppression involves active efforts to inhibit prepotent emotional responses [9,13]. These strategies also affect risk-taking: cognitive reappraisal reduces negative emotional experience and increases adaptive risk-taking, whereas expressive suppression generally does not change risk attitudes. Moreover, habitual reappraisal use is associated with reduced sensitivity to potential losses [14,15]. Experimental studies using paradigms like the Ultimatum Game—a well-established experimental task in which individuals must decide whether to accept or reject monetary offers proposed by another player—demonstrate that cognitive reappraisal can increase acceptance of unfair offers whereas the induction of negative emotional states, such as sadness, has been associated with higher rejection rates [3,12]. ER appears to influence not only social DM but also temporal choice processes, such as the tendency to prefer smaller immediate rewards over larger delayed ones (delay discounting). Reduced impulsivity and a stronger preference for delayed rewards have been linked to cognitive reappraisal, emphasising its role in adaptive DM [16]. Taken together, converging evidence suggests that ER represents a core mechanism through which executive control processes shape adaptive DM [17,18], particularly in emotionally salient or high-stakes contexts.

Executive functions (EF) generally encompass a variety of higher-order cognitive processes, such as planning, working memory, set shifting, error detection and correction, and inhibitory control [19]. They can be categorised into “hot” and “cool” components. Cool EF are typically recruited in abstract, decontextualized and emotionally neutral situations: they include cognitive flexibility, working memory, planning, organisation and inhibitory control and are associated with conscious control, mental effort and analytical processing [20,21]. These processes are recruited for the deliberate self-regulation of emotion [22]. In contrast, hot EF are engaged in contexts characterised by emotional and motivational salience. They involve the processing of reward-related, affective and motivational information and are particularly relevant in ambiguous, uncertain or risky situations [23]. Although conceptually distinct, hot and cool EF rely on partly overlapping brain regions that represent higher-level self-regulatory processing, mediating the regulation of motivation and the integration of emotional and cognitive information [22,24]. Working memory and inhibitory control are particularly important: high working memory capacity supports maintaining and manipulating information while inhibiting distractions, promoting consideration of future consequences; poor inhibitory control increases impulsivity and susceptibility to maladaptive behaviours [25,26] and this could potentially lead to unhealthy choices regarding behaviour. Individuals with poor working memory or inhibitory control may tend to forget about possible negative consequences of their behaviour and engage in riskier substance use and delinquent activities. Therefore, these components of cognitive self-regulatory ability may be important factors influencing whether individuals engage in risk behaviours, by fostering self-regulation over impulsive DM [27]. In addition to rational evaluative processes in DM, such as those associated with EF, somatic and visceral emotional responses to stimulation may also influence DM. The somatic marker hypothesis posits that sympathetic arousal in response to aversive outcomes produces bodily signals guiding choices, a mechanism disrupted by damage to the ventromedial prefrontal cortex. In fact, individuals with damage to this brain area fail to adapt their behaviour in response to anticipated future negative consequences of their actions [17,28,29].

The Iowa Gambling Task (IGT) is widely used to study the integration of emotional-motivational signals with EF, showing that advantageous DM relies on the dynamic interplay of affective reactivity and executive regulation [18,27]. Performance on the IGT has been shown to be negatively associated with impulsivity and sensitivity to reward and punishment [30] and positively correlated with EF abilities [31].

These findings suggest that advantageous DM depends on the integration of emotional–motivational signals with executive control processes. Within this framework, EF serve as a conceptual bridge between ER and DM, allowing for adaptive behaviour by integrating cognitive control with ongoing emotional and motivational states.

### 1.1. Neural Correlates

Converging evidence suggests that ER, DM and EF rely on partially overlapping prefrontal–striatal–limbic circuits. The distinction between “hot” and “cool” EF is consistent with neuroanatomical evidence: ventral prefrontal regions, including the orbitofrontal cortex (OFC), interact with limbic and reward-related structures, supporting the integration of affective and motivational information; whereas lateral regions, like the dorsolateral prefrontal cortex (dlPFC), are linked to distributed cognitive networks playing an important role in cognitive control processes including attention, working memory and action selection [22].

Neuroimaging studies show that ER is supported by a cognitive control network involving dlPFC and ventrolateral prefrontal cortex (vlPFC) that exert a top-down modulation over limbic and striatal areas, in particular the amygdala and the striatum [32,33,34,35]. Specifically, cognitive reappraisal relies on frontoparietal networks that regulate emotional responses by facilitating interaction between prefrontal regions and amygdala [36].

Similarly, DM processes, especially in contexts involving reward and risk, recruit overlapping prefrontal and subcortical regions, including the dlPFC, vlPFC, medial prefrontal cortex (mPFC), OFC, ventral striatum and parietal regions [37,38,39,40,41,42,43]. Evidence suggests that ER can modulate activity within these networks, influencing valuation processes and behavioural choices, particularly through reappraisal-related engagement of prefrontal control regions [44,45].

In addition to prefrontal–limbic interactions, both DM and ER consistently recruit the anterior insula and the dorsal anterior cingulate cortex (dACC). The dACC has been linked to attentional processing and EF, whereas the ventral insula is more closely associated with affective processing [46]. The insula (which extends to the inferior frontal gyrus) and the dACC have been identified as part of a network that supports adaptive DM, particularly in approach–avoidance contexts involving threat minimisation and reward maximisation [47]. Furthermore, meta-analytic evidence suggests that the dACC and the bilateral anterior insula form a domain-general network that is consistently involved in multiple ER strategies [48,49].

Emerging models further distinguish ventral systems (vlPFC and mPFC) for modulating ongoing emotional and behavioural responses, as well as updating outcome expectations and dorsal systems (dlPFC and dmPFC) for attentional control and conflict resolution. The vlPFC is responsible for online response modulation within this framework, while the mPFC is involved in updating expected outcomes, especially when reinforcement contingencies change. Adaptive behaviour is facilitated by dorsal prefrontal regions which enhance the salience of task-relevant or emotionally incongruent representations. These regions support both DM and ER through top-down attentional mechanisms [11].

Overall, these findings emphasise the existence of a shared neurocognitive architecture in which prefrontal regions exert top-down control over affective and reward-related systems.

### 1.2. Clinical Populations

Within this neurocognitive framework, clinical populations characterised by alterations in executive control and affective processing offer a critical opportunity to investigate how these systems break down and influence DM. Clinical populations provide a valuable framework for examining the interaction between ER, EF and DM, as these domains are frequently disrupted across neurological and psychiatric conditions. Despite their heterogeneity, several clinical groups share alterations in executive control, emotional processing and adaptive DM, making them particularly informative for investigating the mechanisms linking these constructs.

Neurodegenerative and neurological conditions, such as dementia and traumatic brain injury, are commonly characterised by executive dysfunctions and difficulties in regulating emotional responses, which may contribute to impaired DM in everyday life contexts [50,51,52]. Neurodevelopmental conditions, such as autism spectrum disorder, often involve atypical socio-emotional processing and altered integration of affective information during DM [53,54,55,56,57]. Substance-related and behavioural addictions are typically associated with heightened reward sensitivity, impulsivity and reduced cognitive control, leading to maladaptive and short-sighted decisions [58,59,60].

Although these conditions differ in aetiology and clinical presentation, they converge in showing disruptions in the interaction between executive and emotional processes underlying adaptive DM. Examining these populations within a unified framework may therefore help clarify whether EF represent a transdiagnostic mechanism linking ER to DM outcomes.

### 1.3. The Present Research

Given the broad and heterogeneous literature on the interactions among ER, EF and DM in clinical populations and the lack of integrative frameworks examining their interplay, a scoping review is particularly appropriate. This approach allows for a comprehensive mapping of existing evidence, the identification of research gaps and the clarification of conceptual frameworks, without restricting the review to narrowly defined interventions or outcomes. Building on this background, the present scoping review specifically focuses on clinical populations, and it aims to highlight trends in the research, identify gaps in knowledge and provide a foundation for future investigations and neurorehabilitation programmes.

## 2. Methods

This scoping review was conducted in accordance with the Preferred Reporting Items for Systematic Reviews and Meta-Analyses extension for Scoping Reviews (PRISMA-ScR) [61,62].

### 2.1. Eligibility Criteria (PCC)

**Population**: Clinical populations with conditions characterised by impairments in emotional, cognitive or DM processes. The selection prioritised conditions with well-established neurocognitive profiles in clinical neuropsychology and cognitive-behavioural frameworks.

**Concept**: Studies investigating at least two of the following constructs: emotion regulation, executive functions and decision-making. Emphasis was placed on the assessment methodologies and measures used to operationalize these constructs (e.g., self-report questionnaires, neuropsychological tests and experimental paradigms such as decision-making tasks).

**Context**: All clinical and research settings were considered. Original empirical studies employing observational or experimental designs were eligible.

**Exclusion criteria**: Reviews, conference abstracts and animal studies were excluded. Studies involving non-clinical populations or investigating only one of the target constructs were also excluded, as were articles not published in English or not appearing in an international peer-reviewed journal. Populations characterised predominantly by acute affective fluctuations were temporarily excluded to allow a more focused investigation of the underlying mechanisms linking EF and ER in DM.

### 2.2. Search Strategy

A literature search was conducted on Google Scholar and PubMed to identify relevant studies investigating the relationships among ER, EF, and DM in clinical populations. The search combined the three core conceptual domains: (1) emotion regulation, (2) executive functions and (3) decision-making processes. These were systematically combined using Boolean operators, together with terms referring to clinical populations and neurocognitive conditions (e.g., dementia, traumatic brain injury, autism spectrum disorder, and addiction-related disorders). Clinical and neurocognitive populations characterised by known executive dysfunctions and emotional dysregulation (such as dementia, traumatic brain injury, autism spectrum disorder and eating disorders) were selected to investigate how these deficits impact decision-making processes across different pathological frameworks.

A representative search string used across databases was:

(“emotion regulation” OR “emotional regulation” OR “affect regulation” OR “emotional dysregulation”)

AND

(“executive function*” OR “inhibitory control” OR “working memory” OR “cognitive flexibility” OR “cognitive control”)

AND

(“decision making” OR “risky decision*” OR “reward-based decision*” OR “intertemporal choice” OR “delay discounting”)

AND

(patient* OR clinical).

The search strategy was iterative, with keywords and combinations refined throughout the screening process to ensure adequate coverage of the relevant literature and to optimise sensitivity across databases. This approach is consistent with established methodologies for scoping reviews, which support iterative refinement of search strategies and the use of supplementary techniques to maximise comprehensiveness. The search was conducted without initial restrictions on publication dates to capture the full extent of the available literature. Additional articles were identified through manual screening of the reference lists of relevant studies.

### 2.3. Study Selection

All articles identified through database searches were imported into the Zotero v9.0.4 reference management software (Corporation for Digital Scholarship, Vienna, VA, USA). The study selection process was conducted in two stages. First, titles and abstracts were screened to identify potentially relevant articles based on the predefined eligibility criteria. In the second stage, the full texts of the articles were assessed for inclusion. The selection process was guided by the a priori-defined eligibility criteria. During the screening of full texts, it became apparent that the included studies could be organised according to four main clinical populations: dementia; traumatic brain injury; autism spectrum disorder; and substance and behavioural addictions. This categorisation was used to structure the presentation of the results and facilitate comparisons between different clinical groups.

### 2.4. Risk of Bias Assessment

Risk of bias was assessed according to study design, following established methodological standards. Case–control studies were appraised using the Newcastle–Ottawa Scale (NOS) [63]; cross-sectional studies with group comparisons were evaluated using the adapted NOS for cross-sectional studies (NOS-xs) [63,64];. Quasi-experimental pre/post designs were evaluated using the Risk Of Bias In Non-randomized Studies of Interventions (ROBINS-I) tool [65]. Finally, randomised controlled trials were assessed with the revised Cochrane Risk of Bias tool (RoB 2) [66].

Because the review included heterogeneous study designs, individual tool outputs were synthesised into a unified summary metric labelled Overall Risk of Bias. This summary rating reflects the proportion of criteria fulfilled within each tool and was categorised as follows: green dot = low risk of bias (≥75% of criteria fulfilled); yellow dot = some concerns (50–74% of criteria fulfilled); and red dot = high risk of bias (<50% of criteria fulfilled). This harmonised approach allows for consistent comparison across studies while preserving the methodological specificity of each appraisal tool.

## 3. Results

A total of sixteen studies met the inclusion criteria and are summarised in Table 1. The distribution across clinical populations was as follows: four studies on dementia [67,68,69,70]; six on traumatic brain injury [51,71,72,73,74,75]; three on autism spectrum disorder [56,76,77]; and three on substance and behavioural addictions [59,78,79]. All included studies investigated at least two of the target constructs, but just two studies examined all three domains [56,79]. A variety of assessment tools and experimental paradigms were used across studies. The Overall Risk of Bias rating is reported in Table 1 for each included study.

### 3.1. Dementia

In neurodegenerative conditions, progressive cognitive decline can compromise the ability to regulate emotion effectively and this can potentially lead to different DM outcomes. EF support goal-directed behaviour and the regulation of emotional responses; therefore, impairments in executive control may exacerbate the difficulty of integrating emotion and cognition, which are necessary for efficient DM and complex adaptive behaviours. Reduced performance on the Iowa Gambling Task, reflected in lower net scores and impaired learning of advantageous deck selections, has been observed in various neurodegenerative disorders, including Parkinson’s disease (PD), Alzheimer’s disease (AD), frontotemporal dementia (FTD) and mild cognitive impairment (MCI) [67,69,80]. As noted by Perach and colleagues (2021), individuals with dementia have limited ER resources, which affects the implementation of antecedent-focused strategies [8,50]. Meanwhile, changes in emotional experience across dementia subtypes may influence response-focused regulation [50,81,82]. While few studies have directly examined the interplay between ER, DM and EF in dementia, several have provided valuable insights.

In a study by Bayard and colleagues (2014), DM on the Iowa Gambling Task was examined in individuals with AD, amnestic MCI and healthy controls [67]. Both clinical groups showed poorer DM than controls, with no difference between AD and amnestic MCI. Notably, poorer DM performance was associated with higher levels of apathy in the action initiation dimension, linking DM impairments to the executive processes that support motivation and goal-directed behaviour [67].

Similarly, Gleichgerrcht and colleagues (2011) found that patients with a behavioural variant frontotemporal dementia (bvFTD) who endorsed utilitarian responses in a personal moral dilemma (the Footbridge dilemma) performed significantly worse on a socio-cognitive task [69]. Importantly, no differences were observed between bvFTD patients who endorsed utilitarian responses and those who did not in executive functioning, global cognition, or affective DM as measured by the Iowa Gambling Task. Taken together, these findings suggest that atypical moral DM in bvFTD is specifically linked to impairments in affective social-cognition rather than to general cognitive or executive deficits [50,69]. However, the study presents a moderate risk of bias due to the use of a small convenience sample without power analysis and the lack of multivariate statistical controls for confounding factors.

Torralva and colleagues (2007) examined DM and social cognition in bvFTD. Compared with controls, patients performed worse on both DM and social cognition tasks [70]. However, performance on the DM task was not correlated with social cognition scores. These results imply that, despite their reliance on overlapping prefrontal networks [18,70,83,84], DM and social cognition may involve partially distinct cognitive processes. Performance on the social cognition task was also associated with executive functioning, indicating that certain aspects of social cognition depend on executive resources [70,85].

Finally, Delazer and colleagues (2009) investigated DM processes through IOWA Gambling Task and the Probability-Associated Gambling Task, in patients with PD and PD dementia [68]. Patients with PD performed similarly to healthy controls, whereas those with PD dementia made significantly riskier and less advantageous choices. These results suggest that impairments in feedback-based learning and emotional processing may affect DM in both groups, while additional deficits in cognitive reasoning specifically characterise PD dementia [68].

### 3.2. Traumatic Brain Injury

Traumatic brain injury (TBI) primarily impacts the frontal lobes and many survivors experience cognitive, social and emotional challenges, such as impaired DM abilities [51,52].

Adlam and colleagues (2017) investigated the performance of survivors of TBI on the Bangor Gambling Task, an emotion-based DM task, where decisions are guided by learning from emotional feedback (wins and losses) rather than explicit probability information [51]. The results showed that only age and group (TBI vs. controls) proved to be significant predictors of overall performance and that TBI survivors made more risky choices than controls, reflected by higher total scores on the task. However, the groups did not significantly differ in performance across blocks, indicating that survivors of TBI and controls showed a similar pattern of change over time and no clear differences in learning the task contingencies. Cluster analysis further identified three subgroups characterised by different decision-making profiles, with both TBI survivors and controls distributed across all clusters. The authors emphasise that individual performance shows considerable variability, suggesting a model based on individual differences to interpret emotional DM in TBI [51].

In a second study by Bonatti and colleagues (2008), TBI patients showed difficulties both in DM under ambiguity (Iowa Gambling Task) and in DM under risk (PAG task and counsel task) compared with healthy controls [71]. TBI patients more frequently selected disadvantageous choices, showed a reduced ability to develop advantageous strategies over time and did not adapt their decisions in response to negative feedback, highlighting deficits in stability and flexibility. Performance on DM tasks was correlated with various components of EF, including cognitive flexibility, planning and psychomotor speed. Difficulty in DM under risk appeared to be linked to inadequate probability estimation, reduced flexibility and cognitive processing capacity. Furthermore, both groups, TBI patients and controls, made more advantageous decisions in a task without feedback compared to a task with feedback, suggesting that a lower emotional load and a simpler presentation facilitate optimal decisions. Overall, the study confirms that TBI patients exhibit specific deficits in DM and highlights the crucial role of EF in supporting advantageous choices [71].

The study by Fogleman and colleagues (2017) investigated DM and reward processing using the modified Iowa Gambling Task in veterans with post-traumatic stress disorder (PTSD), mild TBI or comorbid PTSD/mild TBI [72]. Although overall behavioural performance on the task did not differ significantly between groups, participants with PTSD, mild TBI or comorbid conditions showed greater impulsivity, assessed through the Barratt Impulsivity Scale, and reduced inhibitory control compared with healthy controls. The grey matter volume in the lateral prefrontal cortex (lPFC) was significantly reduced in the clinical groups compared to controls and was associated with altered decision-making patterns on the task, rather than improved performance: in veterans with PTSD it was linked to a relative increase in choices from the “advantageous” decks, whereas in veterans with mild TBI or PTSD/mild TBI comorbidity it was associated with a more general increase in card selections across decks, suggesting a more impulsive response style rather than enhanced decision quality. These findings suggest that structural alterations in the lPFC influence DM strategies and the regulation of impulsive responses, highlighting the role of EF in DM and reward processing [72].

Fonseca and colleagues (2021) compared DM of TBI patients with that of healthy controls using the Iowa Gambling Task, as a measure of hot executive functioning (emotion-based DM), and additionally assessed inhibitory control with the Trial Making Test-B and the Hayling Test, as a measure of cold executive functioning [73]. Although total and block scores on the Iowa Gambling Task did not differ significantly between groups, TBI patients showed a preference for disadvantageous decks and did not exhibit learning curves during the task, suggesting increased risk-taking and impulsive choice. Moreover, the study identified dissociations between the ‘hot’ and ‘cold’ components of EF. Some patients with TBI exhibited selective impairments in ‘hot’ EF, linked to emotionally driven DM, while maintaining normal functioning of ‘cold’ EF. Other patients exhibited partial dissociations between hot and cold components, whilst a minority showed deficits in both hot and cold EF, highlighting the heterogeneity of post-TBI cognitive profiles. This evidence suggests that, although overall performance on the DM may be similar to that of controls, TBI patients exhibit specific impairments in the emotional and strategic integration of decisions (‘hot EF’), while more abstract or neutral inhibitory control (‘cold EF’) may be preserved or dissociated, confirming the considerable heterogeneity of post-TBI cognitive deficits and of the complex interplay between hot and cold EF in DM processes [73].

The study by Stubberud and colleagues (2020) with patients with acquired brain injury, predominantly TBI, examined the relationship between ER and EF in everyday life using questionnaires [74]. ER in particular was assessed through the Brain Injury Rehabilitation Trust Regulation of Emotions Questionnaire, which is a specific instrument assessing ER in patients with acquired brain injury. The results revealed significant correlations between ER and EF, particularly with components related to behavioural and emotional control, indicating a strong overlap between difficulties in ER and executive dysfunctions. Furthermore, ER was significantly associated with symptoms of anxiety and depression. Overall, the results suggest that ER difficulties largely reflect executive deficits in daily life and that the specific measure of ER does not provide substantially additional information compared to assessments of EF and psychological distress [74]. It should be noted that the study presents a moderate risk due to a high non-response rate (49.5%) and the absence of multivariate analysis; furthermore, the primary results are derived exclusively from subjective self-assessment tools.

In line with these findings, evidence from an intervention study by Tsaousides and colleagues (2017) involving individuals with TBI showed that improvements in ER through online training were associated with improvements in executive functioning in daily life, problem-solving and psychological well-being [75]. Difficulties with ER, assessed with the Difficulties in Emotion Regulation Scale, were significantly reduced and these improvements were sustained over time [75]. We must consider that the study presents a critical risk of bias. In fact, the main limitation is the quasi-experimental pre-post design without a control group, which prevents the improvements from being attributed exclusively to the intervention; this is compounded by the subjectivity of the outcomes, which are based solely on self-reports.

Overall, these findings further support the hypothesis of a close interconnection between ER, EF and DM, suggesting that interventions targeting ER may also have positive effects on executive functioning and DM in patients with TBI.

### 3.3. Autistic Spectrum Disorder

Evidence suggests that executive functioning and ER may contribute to social DM difficulties in individuals with autism spectrum disorder (ASD), although their specific roles remain unclear [53,54,55,56,57].

Using the Ultimatum Game, Woodcock and colleagues (2020) found that adolescents with ASD made fewer fair offers than their typically developing peers [56]. This pattern was associated with poorer socio-cognitive abilities (such as emotion recognition, social perception and empathy) but not with measures of executive functioning or ER. In contrast, responder behaviour appeared to be more closely linked to ER processes. Woodcock and colleagues (2020) found that ASD adolescents were less effective at down-regulating negative emotions when faced with unfair offers [56]. It has to be noted that ER was assessed using emotion-specific measures which evaluate children’s ability to manage anger- and sadness-related responses and not ER directly.

Further developmental findings from Jin and colleagues (2020) suggest differences in fair DM. Offers that are not fair are more often accepted by children with high-functioning ASD (HF-ASD) than by their typically developing peers, although this difference becomes less evident during adolescence [76]. In addition, the interaction between behavioural regulation abilities and age is a predictor of acceptance of unfair offers in individuals with HF-ASD. In typically developing individuals, however, such decisions are predicted by working memory and socio-cognitive abilities. Taken together, these findings imply that the ability to make fair decisions in individuals with HF-ASD may be more dependent on intuitive processing and influenced by factors such as age, experience, comorbidity and emotional management [76].

In a study by Sepehri Bonab and colleagues (2025), in children with ASD, an 8-week virtual reality (VR)–based physical exercise programme led to improvements in both EF and ER compared with a sedentary video-gaming control group [77]. Children in the VR condition showed enhanced cognitive flexibility and inhibitory control, alongside better parent-reported ER abilities, whereas no meaningful changes emerged in the control group. The findings suggest that VR-based physical activity may promote adaptive ER in autistic children, potentially through concurrent improvements in executive functioning [77]. However, the study presents a serious risk of bias. The main concern relates to the lack of blinding for the researchers and the parents who provided the data, combined with the use of subjective self-report measures to assess the primary outcome of emotional regulation, which exposes the results to bias stemming from participants’ expectations.

Overall, these findings suggest that ER and broader regulatory processes may play a key role in social DM in individuals with ASD. However, their specific contributions remain unclear.

### 3.4. Substance-Related and Behavioural Addictions

EF and ER are closely associated [86,87,88]: a better performance in one ability is associated with better performance in the other [86] and both abilities suffer alterations in patients with substance use disorder (SUD) [58].

Formiga and colleagues (2021) investigated the relationship between ER assessed with the Emotion Regulation Profile, and EF in SUD patients [78]. Participants with SUD exhibited impairments in EF, particularly in working memory, inhibitory control and cognitive flexibility. They also demonstrated an increased reliance on maladaptive ER strategies compared to non-users, with those who used multiple substances exhibiting the most severe deficits. Poor EF and ER were associated with an earlier onset of substance use, difficulty maintaining abstinence, heightened cravings and maladaptive DM behaviours. In fact, insufficient inhibitory control perpetuates the cycle of craving and relapse; working memory supports goal-directed ER and the maintenance of personal goals; cognitive flexibility enables the adaptation of behaviours that are aligned with goals yet compatible with individual capacities. Authors advanced the idea that preserved working memory and cognitive flexibility enable proactive ER and adaptive DM, whereas their deficit leads to impulsive and automatic behaviours. These findings highlight EF and ER as critical yet malleable targets for intervention in programmes aiming to improve DM in SUD [78]. However, the study presents a moderate risk of bias, compounded by the lack of a power analysis due to the small sample size, the absence of statistical adjustments for age and education level and a marked gender imbalance between the groups.

Similarly, Internet Addiction Disorder (IAD) has been linked to deficits in EF, ER and DM. Interventions such as body awareness psychotherapy have been shown to significantly improved EF (e.g., cognitive flexibility, impulse control and response inhibition), ER and DM performance as assessed by the Iowa Gambling Task, alongside a reduction in the severity of IAD. While DM abilities improved following the intervention, mediation analyses indicated that only improvements in EF and ER significantly mediated the relationship between body awareness psychotherapy and the reduction in IAD. These findings suggest that improved DM may be a consequence of enhanced executive and ER processes rather than a primary mechanism [79]. This study presents a moderate risk of bias, as there are some concerns regarding the use of self-report measures for the primary outcome of internet addiction, combined with the fact that the researchers supervised the data collection in an open manner, which could influence participants’ subjective perception of improvement.

These findings highlight EF and ER as key targets for preventing and treating addictive behaviours; in fact, strong EF abilities may protect against the early onset of substance use [89], while effective ER skills support abstinence during and shortly after treatment [90]. Overall, ER is crucial for the development, severity, treatment and prognosis of SUD and cognitive deficits exacerbate addiction outcomes [91].

Eating disorders and obesity are characterised by pathological eating behaviours influenced by emotional and neuropsychological impairments on measures of cognitive flexibility, central coherence, DM and aberrant reward processing [59,60]. Negative affect can trigger maladaptive eating behaviours and EF are crucial for regulating these choices [92,93]. Segura-Serralta and colleagues (2018) found that patients with eating disorders and obesity showed impairments in DM and central coherence, and that affect (positive and negative) and cognitive variables significantly predicted ER, suggesting that EF mediate the impact of emotions on food-related DM [59]. Clinical participants tended to make decisions based on immediate rewards despite long-term negative consequences, highlighting how difficulties in integrating cognitive and emotional information may reinforce maladaptive eating patterns [59].

## 4. Discussion

The aim of this scoping review is to map the evidence on the relationship between EF, ER and DM across different clinical populations, with the intention of clarifying how these domains interact. It is worth noting that the depth of discussion dedicated to each clinical population varies across the following sections. Rather than a stylistic choice, this asymmetry directly reflects the current density and historical trajectory of the literature. The findings suggest that DM does not reflect an isolated function but rather emerges from the dynamic interaction and integration between EF and ER. Across studies involving different clinical populations, impairments in ER were frequently associated with deficits in executive control and with maladaptive DM, particularly in contexts involving uncertainty, reward evaluation and social interactions. This pattern indicates that EF may play a central role in integrating emotional responses into goal-directed decisions. However, this integration is probably an example of two processes working together in both directions at the same time. This relationship does not show that one process depends on the other. Instead, the two systems work together to regulate emotional responses and cognitive processing during DM process.

Another finding observed in the reviewed literature is that executive dysfunctions seem to limit the effective implementation of ER strategies during DM. Deficits in inhibitory control, cognitive flexibility and working memory were consistently associated with a preference for immediate rewards, reduced sensitivity to feedback, and difficulties adapting behaviour over time [59,71,72,78,79]. These findings support the idea that EF enable individuals to modulate emotional responses, maintain long-term goals and integrate affective and cognitive information when making decisions. Conversely, preserved EF appear to facilitate proactive ER strategies and more advantageous DM [77,79], further supporting the idea that EF and ER work together in an interactive way to support adaptive DM processes.

The distinction between ‘hot’ and ‘cold’ EF provides further understanding of the mechanisms linking ER and DM. One revised study reported selective impairments in emotionally driven DM, despite relatively preserved performance in neutral executive tasks. This suggests that difficulties primarily arise from the altered integration of affective signals and executive control, rather than from general cognitive deficits [73]. This pattern provides further support for the idea of the dynamic interaction during DM, with engagement of partially overlapping neural systems involved in both executive and affective regulation.

Another consistent pattern concerns reward sensitivity and feedback-based learning. Across various clinical conditions, maladaptive DM was often characterised by increased impulsivity, reduced learning from negative outcomes and a preference for short-term rewards despite the long-term negative consequences [59,72,73,78]. These behaviours appear to reflect the combined impact of heightened emotional reactivity and reduced executive control. This suggests that EF may modulate the influence of emotional responses on reward-based DM. The preference for immediate rewards highlights impaired self-regulation, where diminished executive control and difficulty managing emotional states contribute to maladaptive DM.

Importantly, intervention studies further support this interpretation, showing that improvements in ER are accompanied by gains in executive functioning and enhanced DM performance. This indicates that EF may represent a modifiable mechanism underlying adaptive decisions [75,77,79].

Taken together, these studies emphasised that the relationship between ER, EF and DM should not be conceptualised as a unidirectional pathway, but rather as a dynamic process. In this context, both EF and ER can be viewed as top-down control mechanisms operating in parallel to regulate and integrate emotional and cognitive inputs which thus constitute a complex mechanism of self-regulation for efficient DM. It is important to note that this integrative framework is a tentative hypothesis, as the current evidence base does not allow us to definitively characterise the directionality and specificity of the interactions between EF and ER. Instead, the proposed model is a preliminary synthesis that aims to organise heterogeneous findings into a coherent explanatory structure.

**Figure 1:**
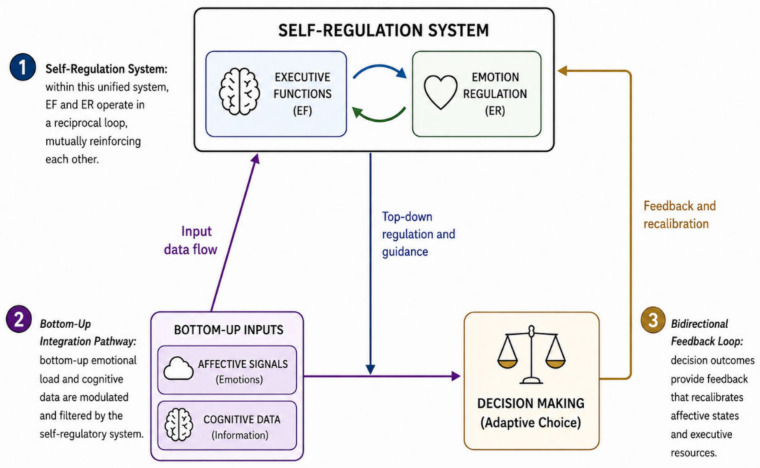
Proposed integrative hierarchical framework of self-regulation and decision-making. The model illustrates the synergistic interaction between: (1) Self-Regulation System, characterised by a bidirectional coupling between Executive Functions (EF) and Emotion Regulation (ER); (2) Bottom-Up Integration Pathway, where incoming affective signals and cognitive data are filtered and modulated by top-down self-regulatory system; and (3) Bidirectional Feedback Loop, where Decision-Making (DM) outcomes dynamically loop back to recalibrate subsequent affective states and executive resources.

To go beyond a descriptive synthesis, we propose a testable integrative model (Figure 1) based on a hierarchical architecture where top-down regulation and bottom-up inputs converge to shape DM.

This framework is structured around a higher-level ‘Self-Regulation System’, in which EF and ER interact bidirectionally to maintain cognitive control and emotional scaffolding. At the lower level, a bottom-up stream delivers affective signals (emotions) and cognitive data. Within this architecture, the synergistic interplay within the Self-Regulation System acts as the primary integration hub, through the synergistic interplay of EF and ER, which co-act to drive DM processes with three distinct yet complementary mechanistic pathways:

1. System of Self-Regulation: within this single, unified self-regulatory system, EF and ER operate as parallel, mutually reinforcing mechanisms. Cognitive control and emotional regulation interact within a reciprocal loop, where executive resources dynamically support the deployment of adaptive ER strategies, while successful emotion regulation simultaneously preserves the cognitive capacity needed for efficient processing, collectively shaping subsequent choice behaviour.
2. Bottom-Up Integration Pathway: as bottom-up emotional load and cognitive data enter the stream, the top-down self-regulatory system functions as a buffer. It modulates and filters the impact of these bottom-up affective signals, including emotions, arousal states and autonomic nervous system responses, preventing emotional reactivity from overwhelming adaptive choice selection.
3. Bidirectional Feedback Loop: the final output of this integration determines DM performance, bridging the gap between standardised laboratory paradigms (such as the Iowa Gambling Task) and real-life adaptive choices. Crucially, these decision outcomes do not represent a terminal point; they provide dynamic feedback that loops back to recalibrate subsequent affective states and executive resource allocation.

Importantly, these pathways are not mutually exclusive but operate in tandem as part of a dynamic, integrated system. By articulating these specific interaction pathways, this framework moves from a tentative hypothesis to an operational roadmap, providing a clear theoretical foundation for targeted empirical testing and clinical application.

The next important step within this framework is to develop and implement experimental approaches that can directly test this integrative framework between EF and ER as top-down regulatory processes during DM. This will clarify their dynamic relationship and the underlying mechanisms.

Indeed, despite these converging findings, there is limited literature on directly testing this dynamic framework, as only a few studies have assessed ER, EF and DM simultaneously. Two intervention studies conducted in clinical populations appear to be moving in this direction. One of these studies targeted executive functioning and emotion regulation through a body awareness psychotherapy intervention in individuals with IAD. The other study used a virtual reality–based physical exercise programme aimed at enhancing executive functioning and ER in children with ASD [77,79].

Furthermore, it should be noted that some of the studies included in this review (e.g., [67,68,69,70,76]) examined affective states, such as depression and anxiety, as well as socio-cognitive abilities, such as empathy or theory of mind. These provide only indirect insights into ER and did not use instruments designed to explicitly assess the construct. Nevertheless, these studies were retained as they directly examined both DM and EF, thereby meeting the predefined inclusion criteria. It is important to emphasise that constructs such as depression and apathy reflect emotional experience or motivational deficits rather than the strategies individuals use to regulate their emotions. This distinction is fundamental and may partly explain the inconsistencies observed across studies. More generally, while several studies have proposed ER as a mechanism linking EF and DM, ER is often not assessed directly using validated measures, but rather inferred or theoretically hypothesised. This highlights a significant gap in the literature, as the absence of explicit assessment limits our ability to draw definitive conclusions about ER’s role.

Furthermore, the substantial heterogeneity in task paradigms and operationalisations of ER and EF limits the comparability of studies and hinders the identification of specific mechanisms. Specifically, the included literature relies on a combination of self-report questionnaires and behavioural task paradigms. While behavioural tasks capture real-time, performance-based cognitive and emotional processing, self-report tools reflect perceived, subjective behavioural patterns. These methodological differences imply that the instruments may measure distinct underlying constructs or levels of awareness, which makes direct comparisons difficult. Beyond this broad methodological divide, there is a lack of standardisation in the tasks themselves. Distinct studies use different cognitive and emotional paradigms instead of a shared, validated set of tests. As these diverse tasks and questionnaires impose different cognitive loads and access distinct underlying mechanisms, direct cross-study comparisons are limited. Consequently, a high degree of interpretive caution is required, as these methodological inconsistencies emphasise the necessity of integrative approaches that directly test theoretical models linking emotional and executive regulation to DM.

Overall, this scoping review lends support to a model in which EF play a pivotal role in bridging the gap between ER and DM across clinical populations. Disruptions in executive control may impair the ability to regulate emotional responses, leading to short-sighted and maladaptive decisions, particularly in emotionally salient contexts. In this view, DM emerges as the outcome of the continuous interaction between EF and ER processes, which jointly modulate the influence of bottom-up affective signals through top-down control mechanisms. This perspective has important clinical implications, suggesting that interventions targeting executive functioning and ER simultaneously could improve DM abilities promoting adaptive behaviour in daily life. Overall, the current evidence supports a preliminary and tentative framework, which requires further empirical validation. Given the complexity of the interactions between EF, ER and DM, current findings should be interpreted as indicative rather than conclusive. This highlights the need for more systematic and experimentally controlled investigations. Future research should adopt longitudinal and experimental designs, assessing ER, EF and DM within the same framework. Furthermore, it should directly test mediation models to clarify the mechanisms linking emotional and executive regulation to adaptive DM outcomes.

### 4.1. Transdiagnostic Mechanisms vs. Disorder-Specific Phenotypes

Including heterogeneous clinical populations in this review enables us to distinguish between common processes and manifestations specific to particular disorders. Mechanisms that recur across diverse clinical conditions may represent shared neurocognitive vulnerabilities, whereas patterns confined to specific disorders may reflect unique pathological or cognitive characteristics.

Across diagnostic categories, the most significant convergent feature is impaired DM; this impairment manifests as a marked preference for immediate rewards despite long-term negative consequences, coupled with reduced sensitivity to environmental feedback and punishment. This trend has been consistently observed in patients with dementia, TBI, ASD and addictions [59,68,71,72,73,76,78]. From an internal processes perspective, the common feature is the absence of ‘emotional scaffolding’ for cognitive processes: affective signals are not properly integrated with EF, which prevents effective top-down regulation [67,69,73,80,89,90]. Inhibitory control emerges as a key process in linking emotional states to DM outcomes. This functional overlap is underpinned by a shared neural architecture involving prefrontal–striatal–limbic circuits, with critical nodes in the dorsolateral prefrontal cortex (dlPFC), ventrolateral prefrontal cortex (vlPFC) and insula [22,32,35].

Regarding the specific mechanisms underlying different disorders, this review proposes the ‘self-regulatory system’ as a cross-cutting factor that may be compromised in different ways depending on the condition.

In cases of addiction and eating disorders, a ‘bottom-up’ breakdown hypothesis may be applicable due to excessive emotional load. This breakdown may be caused by the overstimulation of affective signals. Hypersensitivity to reward and cycles of craving could generate excessive emotional stimulation, which would overwhelm the ‘buffer’ of the self-regulatory system. In such cases, DM processes would fail because emotional impulses would be too intense to control, resulting in impulsive and automatic behaviour [59,78,79].

In cases of dementia (such as AD, FTD and PD), we could consider a ‘top-down’ breakdown hypothesis due to the weakness of the control mechanisms. The system appears to break down due to a depletion of the executive resources required to implement regulatory strategies. In this context, failure may be more structural in nature, with a reduced capacity for initiative and goal maintenance (often associated with apathy) making proactive strategies such as reappraisal more difficult to use [50,67,68].

Patients with TBI represent a complex and heterogeneous group because, depending on the location and extent of the injury, they may exhibit a wide range of cognitive, emotional and behavioural deficits. Previous reviews have confirmed that these patients often have severe deficits in DM [94]. For example, structural damage to the lateral prefrontal cortex (lPFC), which is typically associated with impairments of a more ‘executive’ and regulatory nature, could selectively impair the integration of affective signals in the DMN, leaving abstract cognitive abilities intact but making patients less able to regulate their responses to salient stimuli [72,73]. Furthermore, it has been observed that a reduced emotional load facilitates more beneficial decisions in these patients, suggesting that difficulties arise specifically when the integration of affective feedback is required [71]. In contrast to the regulatory and operational deficits of the lPFC, lesions to the ventromedial prefrontal cortex (vmPFC) selectively affect the emotional sphere by preventing the generation of emotional signals (somatic markers) needed for decision-making. In such cases, a dual dissociation is observed: patients exhibit severe decision-making impairment yet retain intact ‘cold’ cognitive functions, such as working memory, attention, and general intellectual abilities [18].

Finally, in ASD, the disruption to the system appears to be linked not so much to a deficit in control, but rather to difficulty integrating complex socio-emotional information, such as empathy and theory of mind, into DM, outlining hypotheses regarding the specificity of social cognition [56,76].

In conclusion, while the ‘Self-Regulation System’ is a common transdiagnostic framework, its vulnerabilities appear to be condition-specific. It is crucial to recognise whether DM failure stems predominantly from an inability to exercise control (top-down) or from excessive emotional reactivity (bottom-up), to guide future research towards the development of targeted and personalised neurorehabilitation interventions.

### 4.2. Limitations

This review has some limitations. Firstly, the small number of studies and their heterogeneity in terms of populations, methodologies and assessment tools makes it difficult to draw definitive conclusions. Secondly, only a few studies have examined all three constructs simultaneously, which limits our understanding of their interactions. Therefore, as this is a scoping review, the aim was to provide a broad overview of the literature rather than a quantitative synthesis. Therefore, it is not possible to formally assess effect sizes or the strength of associations. Finally, in line with the neuropsychological focus of this work, this review intentionally prioritised conditions deeply rooted in clinical neuropsychology and cognitive behavioural frameworks (e.g., TBI, dementia, ASD and addictions). However, we recognise that the scope of the review is limited by these methodological choices. The variability observed in populations such as those with TBI suggests that our framework requires further testing. Future reviews that extend these boundaries to severe psychopathological conditions, such as bipolar disorder and borderline personality disorder, will be invaluable in testing whether the proposed integrated self-regulation framework is generalisable to a broader clinical spectrum, characterised by unique dynamics between acute affective fluctuations and cognitive control.

### 4.3. Future Directions

From a neurocognitive perspective, these findings support models emphasising the role of prefrontal–limbic circuits in supporting an integrated self-regulatory system underlying DM. Disruptions in these networks are likely to reflect alterations in this dynamic interaction and integration, rather than a simple reduction in emotional input or a deficit in top-down cognitive control. Importantly, both EF and ER represent potentially modifiable processes, making them promising targets for biofeedback and neuromodulation-based interventions.

On the one hand, biofeedback could play an important role in this context for at least two reasons. First, in terms of assessment, certain indices such as heart rate variability (HRV) or blood volume pulse (BVP), which are considered (parasympathetic) indices of top-down self-regulation (see, e.g., [95,96]), have already been linked to improved executive functioning [97,98] and better emotional regulation abilities [99,100] and are therefore considered potential—in vivo and non-invasive—transdiagnostic biomarkers of mental health [101]. Conversely, alterations in the bottom-up peripheral (sympathetic) signal of electrodermal activity (EDA) have been found, for example, in TBI patients with impaired DM [102]. The possibility, therefore, of considering autonomic balance (sympathetic system versus parasympathetic system) could represent an important transdiagnostic biomarker that provides insight into how bottom-up physiological information and activations can be integrated and modulated through top-down control (as indicated by HRV indices). When mapped onto our framework, these autonomic metrics offer a direct, empirical window to investigate the internal dynamics of the Bottom-Up Integration Pathway.

Secondly, these indices can also be utilised in rehabilitation through specific biofeedback protocols that can help individuals—using specific protocols—self-regulating their autonomic indices, potentially thereby also affecting their self-regulatory system (executive functioning and emotional regulation abilities) and thus leading to more effective DM. From a mechanistic standpoint, such interventions serve as an ideal paradigm to empirically validate the Bidirectional Feedback Loop, demonstrating how stabilising peripheral autonomic reactivity can shield cognitive resources from bottom-up overload and indirectly facilitate more adaptive decision-making performance.

On the other hand, as seen in Section 1.1., ER and DM share dorsomedial, dorsolateral, ventrolateral and medial regions of the prefrontal cortex [11]. Due to these characteristics, brain stimulation to these areas using neuromodulation tools can influence ER and DM by modulating the interaction in the brain circuits [103].

In a review by Choi and colleagues (2016) it has been shown that both repetitive Transcranial Magnetic Stimulation (rTMS) and Transcranial Direct Current Stimulation (tDCS) improve ER and DM abilities by modulating top-down regulation [104]. With respect to ER, rTMS has been shown to influence attentional and affective processing of emotional stimuli, as well as autonomic responses, with additional evidence suggesting that cerebellar stimulation may impact mood-related processes. tDCS studies, which are more heterogeneous in scope, indicate region-specific effects on emotional arousal, regulation of negative affect, emotional reactivity to aversive stimuli and ruminative self-referential thinking. In the domain of DM, rTMS has been associated with changes in delay discounting, food-related choices, moral judgement and blameworthiness decisions, as well as in executive domains such as visuospatial attention, working memory, perception and visuomotor performance. Similarly, tDCS has been reported to influence risk-taking, impulse control, maladaptive DM and value-based choice, in addition to broader cognitive processes including dual-task performance and model-based learning [104].

Clinical findings further reinforce this interpretation. For example, bilateral tDCS targeting the dorsolateral prefrontal cortex (dlPFC) in individuals with borderline personality disorder led to improvements in executive functioning and increased use of cognitive reappraisal strategies, suggesting that modulation of prefrontal control networks may reduce emotion dysregulation [105]. When mapped onto our model, neuromodulation tools like tDCS and rTMS serve a dual purpose: they represent a valuable experimental approach to causally validate the architecture of our framework and a promising symptom-targeted intervention for clinical populations. By applying stimulation over the dlPFC, researchers can causally enhance prefrontal activity during standardised DM tasks. This approach allows investigators to examine whether boosting executive resources simultaneously enhances emotion regulation capacities (to test the strength of the internal dynamics of the Self-Regulation System) and whether it increases a person’s resilience to emotional biases while making choices (validating the top-down buffer capacity). Economically and practically, tDCS offers practical advantages in terms of ease of application, tolerability and relatively low cost, making it particularly suitable for large-scale clinical research. Ultimately, by explicitly identifying which regulatory components these interventions target, our framework supports the future development of integrated, dual-target protocols (e.g., combining tDCS for top-down enhancement and biofeedback for bottom-up stabilisation) tailored to the specific breakdown of different clinical cohorts. Given its capacity to modulate prefrontal control networks, tDCS represents a promising tool not only as a valuable experimental approach to elucidate shared neural mechanisms underlying executive control, ER and DM, but also as a promising symptom-targeted intervention for disorders marked by emotional dysregulation and maladaptive choice behaviour. However, further research is needed to establish long-term clinical efficacy and to define optimal stimulation protocols [104]. Future research should adopt more integrative and ecologically valid paradigms, assessing ER, EF and DM within the same experimental framework. Longitudinal designs may also help to clarify the directionality of these relationships.

## 5. Conclusions

In conclusion, this scoping review emphasises the importance of moving beyond reductionist explanations of DM and adopting an integrative, system-level approach. ER and EF should be conceptualised as interacting components of a broader self-regulatory system shaping decision behaviour. Developing this framework will be crucial for advancing theoretical understanding and creating more effective and targeted interventions for different clinical populations.

## Figures and Tables

**Table 1:** Summary of the included studies examining the relationships among DM, ER and EF across clinical populations, including sample characteristics, assessment measures and overall risk of bias.

Study	Participants	Study Design	DM	ER	EF	Overall Risk of Bias
**Dementia**						
Torralva et al., 2007 [70]	20 patients early/mild behavioural variant frontotemporal dementia (bvFTD) (9 females; age 67.2 ± 8.1) 10 controls (6 females; age 63.5 ± 5.8).	Cross-sectional, observational case–control design	IOWA Gambling Task (IGT) computerised version.	X	Verbal fluency Raven-coloured progressive matrices (NCS Pearson, San Antonio, TX, USA); Digit span backwards; Trail Making Test-B; Letters and numbers ordering subtest from the Wechsler Adult Intelligence Scale (WAIS; NCS Pearson, San Antonio, TX, USA); Wisconsin card sorting test (WCST; (Psychological Assessment Resources, Lutz, FL, USA), modified version; Frontal assessment battery (FAB).	●
Delazer et al., 2009 [68]	19 demented idiopathic Parkinson’s disease (PD) patients (11 females; age 72.2 ± 5.1) 20 non-demented idiopathic PD patients (5 females; age 68.5 ± 5.9) 20 healthy participants (17 females; age 71.3 ± 3.5).	Cross-sectional, observational case–control design (multi-group)	IGT; Probability-Associated Gambling Task (PAG).	X	Trail Making Test-B; Odd-Man-Out, (OMO); Just for patients: FAB; Tests of categorical verbal fluency (animals/min, CERAD); Planning (CLOX1).	●
Gleichgerrcht et al., 2010 [69]	22 patients with early/mild stages of bvFTD with frontal atrophy on Magnetic Resonance Imaging (11 females; age 71.3 ± 5.9).	Cross-sectional, observational design (within-cohort comparison)	Moral Behaviour Inventory; Footbridge dilemma; IGT.	X	Backward digit span task; Trail Making Test-B; Phonological (letter “P”) fluency; Modified version of the WCST; INECO Frontal Screening.	●
Bayard et al., 2014 [67]	20 Alzheimer’s disease patients (12 females; 80.9 ± 5.4) 20 amnestic Mild Cognitive Impairment patients (11 females; 78.25 ± 6.9) 20 healthy controls (11 females; 73.5 ± 6.7).	Cross-sectional, observational case–control design (multi-group)	IGT.	X	Trail Making Test-B; Hayling Test; Updating Memory Task.	●
**Traumatic brain injury (TBI)**						
Bonatti et al., 2008 [71]	21 TBI patients (3 females; 34.5 ± 11.8) 20 controls (14 females; 31.9 ± 13.1).	Cross-sectional, observational case–control design	IGT; PAG; Counsel version of PAG task.	X	Digit span backward; Trail Making Test-B; Verbal fluency [animals/min, s-words/min of the Regensburger Wortflu ssigkeitstest (RWT)]; Cognitive estimation [Test Zum Kognitiven Schätzen (TKS)]; Planning; OMO; Go-No Go computerised version; Graded Difficulty Arithmetic Examination.	●
Fonseca et al., 2012 [73]	16 TBI patients 4 females; mean age 37.31 ± 13.65); 16 controls (7 females; 32.88 ± 13.09).	Cross-sectional, observational case–control design	IGT computer-based version adapted to the southern Brazilian population.	X	Trail Making Test-B; Hayling Test.	●
Adlam et al., 2017 [51]	30 survivors of TBI (5 females; mean age 34); 39 healthy controls (22 females; mean age 38).	Cross-sectional, observational case–control design	Bangor Gambling Task (BGT).	X	Digit span and letter−number sequencing WAIS-III; Modified Six Elements (6 Elements) subtest of the Behavioural Assessment of the Dysexecutive Syndrome (BADS; (Pearson Clinical, London, UK); Dysexecutive Syndrome Questionnaire (DEX).	●
Fogleman et al., 2017 [72]	21 Veterans diagnosed with post-traumatic stress disorder (PTSD) (16 males; 29.95 ± 4.97) 18 Veterans diagnosed with mild TBI (18 males; 29.22 ± 5.08) 26 Veterans diagnosed with co-occurring PTSD/mild TBI (23 males; 30.23 ± 5.26) 23 Veteran controls without PTSD or mild TBI diagnoses (19 males; 30.61 ± 6.72).	Cross-sectional, observational case–control design (with structural neuroimaging)	IGT modified version.	X	Barratt Impulsivity Scale.	●
Tsaousides et al., 2017 [75]	91 individuals with TBI and deficits in ER (51 females; 47.08 ± 11.84).	Quasi-experimental, pre-/post within-subject design (longitudinal)	X	Difficulties in Emotion Regulation Scale (DERS).	Problem Solving Inventory; Social Problem Solving Inventory-Revised: Short Form; Dysexecutive Questionnaire.	●
Stubberud et al., 2020 [74]	70 patients with verified acquired brain injury and self-reported executive difficulties in daily life at least 6 months post-injury (38 males; 42.89 ± 12.96).	Cross-sectional, observational design (within-cohort/associative)	X	Brain Injury Rehabilitation Trust Regulation of Emotions Questionnaire.	Tower Test; Behaviour Rating Inventory of Executive Function Adult Version.	●
**Autistic spectrum disorders**						
Woodcock et al., 2019 [56]	20 adolescents with autism (4 females; mean age 13.3) and a matched typical reference sample (sample size not specified).	Cross-sectional, observational case–control design	Ultimatum Game (UG).	Children’s Anger Management Scale (CAMS) Children’s Sadness Management Scale (CSMS).	Behaviour Rating Inventory of Executive Function (BRIEF; Psychological Assessment Resources, Lutz, FL, USA).	●
Jin et al., 2020 [76]	31 children and adolescents with high functioning autism spectrum disorder (4 females; 9.01 ± 2.69) 38 children and adolescents with typical development (5 females; 9.72 ± 2.76).	Cross-sectional, observational case–control design	Ultimatum Game (UG).	X	BRIEF.	●
Sepehri Bonab et al., 2025 [77]	40 boys diagnosed with autism spectrum disorder were randomly assigned to two groups: 20 to a virtual reality intervention group 8.89 ± 0.9) 20 to a control group (8.94 ± 1.2).	Quasi-experimental, pre/post-test control group design	X	Emotion Regulation Checklist (ERQ).	WCST; Flanker task test.	●
**Substance-related and behavioural addictions**						
Segura-Serralta et al., 2018 [59]	39 healthy participants (30.36 ± 11.95) 33 patients with obesity (45.39 ± 12.35) 30 patients with restricting eating disorder (22.80 ± 8.05) 18 purging eating disorder patients (25.50 ± 9.02).	Cross-sectional, observational case–control design (multi-group)	IGT computerised version.	Spanish adaptation of the DERS.	WCST;.	●
Formiga et al., 2021 [78]	60 non-substance use disorder but could be alcohol users or tobacco (38 females; 27 ± 11) 51 with alcohol use disorder only or alcohol and tobacco use disorder (17 females; 36 ± 11) 19 with multiple substance use disorder, including at least one of the illicit substances, named polysubstance users (5 females; 33 ± 8).	Cross-sectional, observational multi-group design	X	Emotion Regulation Profile (ERP).	Trail Making Test-B; Stroop test, part C; The Digits subtest in the Wechsler Intelligence Scale for Children 3rd edition (WISC-III; NCS Pearson, San Antonio, TX, USA).	●
Fallah et al., 2025 [79]	60 university students with internet addiction disorder: 30 experimental group (19 females; 25.07 ± 3.10); 30 control group (13 females; 24.50 ± 3.10).	Randomised Controlled Trial (RCT)	IGT.	DERS.	Stroop effect task.	●

**DM** = Decision-Making; **ER** = Emotion Regulation; **EF** = Executive Functions. **Overall Risk of Bias Legend:** ● **Green** = Low risk of bias (≥75% of criteria fulfilled); ● **Yellow** = Some concerns (50–74% of criteria fulfilled); ● **Red** = High risk of bias (<50% of criteria fulfilled).

## Data Availability

No new data were created or analysed in this study. Data sharing is not applicable to this article.
